# Influence of Branching on the Configurational and Dynamical Properties of Entangled Polymer Melts

**DOI:** 10.3390/polym11061045

**Published:** 2019-06-14

**Authors:** Alexandros Chremos, Jack F. Douglas

**Affiliations:** Materials Science and Engineering Division, National Institute of Standards and Technology, Gaithersburg, MD 20899, USA

**Keywords:** polymers, entanglement, branching, packing length, translational diffusion coefficient, hydrodynamic radius, hyperuniformity, decoupling, packing length, glass-formation

## Abstract

We probe the influence of branching on the configurational, packing, and density correlation function properties of polymer melts of linear and star polymers, with emphasis on molecular masses larger than the entanglement molecular mass of linear chains. In particular, we calculate the conformational properties of these polymers, such as the hydrodynamic radius $R_{h}$, packing length *p*, pair correlation function g(r), and polymer center of mass self-diffusion coefficient, *D*, with the use of coarse-grained molecular dynamics simulations. Our simulation results reproduce the phenomenology of simulated linear and branched polymers, and we attempt to understand our observations based on a combination of hydrodynamic and thermodynamic modeling. We introduce a model of “entanglement” phenomenon in high molecular mass polymers that assumes polymers can viewed in a coarse-grained sense as “soft” particles and, correspondingly, we model the emergence of heterogeneous dynamics in polymeric glass-forming liquids to occur in a fashion similar to glass-forming liquids in which the molecules have soft repulsive interactions. Based on this novel perspective of polymer melt dynamics, we propose a functional form for *D* that can describe our simulation results for both star and linear polymers, covering both the unentangled to entangled polymer melt regimes.

## 1. Introduction

Polymers play an important role in materials in everyday life, including film packaging, the molded parts of furniture, airplanes, and automobiles, as well as, diverse tools and devices for industry and the medical sciences. The usefulness of polymers is due to the many advantages of polymeric materials in comparison to metals, e.g., low weight, corrosion resistance, thermally and electrically insulating properties, along with lower processing and maintenance costs. A central feature of polymers is that material properties can be greatly influenced by the molecular characteristics, in addition to the chemical nature of the monomers [1,2,3], so that polymeric materials are literally a “plastic” form of matter.

Two basic topological molecular characteristics of polymers are chain length and topological interactions arising from repulsive interpolymer interactions. For example, when the length of the polymer chains in a melt is relatively short, the resultant bulk material displays common features of low molecular mass ($M_{w}$) materials, such as propensity to form brittle powders. However, when the length of the polymer chains is relatively long, then the topological interactions between the polymer chains result in “entangled” structures that greatly restrict chain motion and augment the transmission of mechanical stresses within the material. It is not really clear at present to what extent entanglement represents a tendency of chains to be localized by surrounding chains versus the extent that interchain interactions give rise to collective chain motion in the form of dynamic clusters of polymers. Branching normally reduces polymer entanglement since polymer conformations become more compact, while stiffer chains tend to be more entangled for a given chain length, provided the polymers are not so stiff that liquid crystalline ordering occurs instead.

Given the long history of polymer science, there are several proposed polymer models that have been introduced to describe the dynamics of the polymers in the melt state [1,2,3]. The Rouse model [4] is often used to describe the dynamics of low molecular mass polymers. This model predicts that the shear viscosity, η, scales as the square of the polymer radius of gyration [5], i.e., $\eta ∼R_{g}^{2}$, which reduces to $\eta ∼M_{w}$. This scaling relation arises from the “Flory theorem” indicating that excluded volume interactions in the melt are screened, resulting in polymer chains having configurations effectively equivalent to random walk chains [6,7], i.e., $R_{g}∼M_{w}^{1/2}$. The Rouse model is broadly consistent with experimental findings [8,9,10], but several studies point to deviations from the Rouse model [9,11,12,13]. When the polymer chains become long enough they enter into the so called “entangled” regime, defined empirically by a stronger scaling in η with $M_{w}$ [5], i.e., $\eta ∼M_{w}^{3.4}$. The reptation model [2,14,15], and its various modifications, have found success in rationalizing the emergence of this change of mass scaling for η and the polymer diffusion coefficient, *D*. Currently, the reptation class of models, emphasizing chain localization by surrounding polymers, dominates the modeling of linear polymer melts, but several inconsistencies remain [16,17,18]. For the modeling of star polymer melts, the concept of arm retraction [19,20] of invoked to rationalize their dynamics, but as in the case of reptation for linear chains, there are many open questions [18,21]. An additional source of concern about these models is their emphasis on the role of polymer topology in relation to the polymer motion within a background matrix of other fixed chains. This type of phenomenological modeling leads to an increasing number of different mechanisms of polymer dynamics for each topology. In our view, this proliferation of models only highlights the need for a unifying framework for understanding polymer melt dynamics.

While experiments have been instrumental in testing the macroscopic predictions of polymer theories, computer simulations can be utilized to probe the microscopic structure of polymers. Atomistic simulations are often employed to probe the structure and the dynamics of polymers in solution and in melt state [22,23,24,25,26,27,28,29,30,31]. Their value lies in matching the properties of specific monomers and by comparison of conformational and dynamical properties between different monomer chemistries to gain insights on how to design polymeric materials with optimal material properties. A significant limitation of atomistic simulations is that are computationally expensive as the trajectory of every atom needs to be calculated. Coarse-graining provides accessibility to larger length scales and longer time scales, i.e., reducing the computational costs, by losing detailed information associated with the structure of the target monomer [32,33,34,35,36,37]. A good coarse-grained model is one that balances these two effects. Multiple coarse-grained approaches have been proposed in the past. We focus on a well established coarse-grained bead-spring model with thermodynamics consistent model that provides access to the long time scales necessary to probe the dynamics in the entangled regime.

In a previous study [38], we have successfully utilized established hydrodynamic models to describe the polymer dynamics of unentangled polymers under the assumption that continuum hydrodynamic theory should be applicable to molecular and polymer fluids. Our aim here is to expand upon our previous work to address the entangled polymer melt regime. We focus, mainly on linear chains and regular stars, but we also briefly discuss unknotted ring and bottlebrush polymers. We investigate the packing properties of entangled linear chains and make comparisons with branched polymers of different degrees of branching having same molecular mass. We also probe how a given polymer interacts with its environment. We calculate the hydrodynamic radius $R_{h}$ with the use of a path integration algorithm ZENO [39] and the self-diffusion of the polymer center of mass, *D*. We propose a functional form for *D* based on $R_{h}$ that can provide a description for unentangled and entangled polymer chains, as well as, for regular star polymers. Our findings suggest a tentative unifying framework of polymer melt dynamics that is agnostic about the role of topology on the nature of polymer diffusion.

Our paper is organized as follows. Section 2 contains details of the model and simulation methods. Results of the packing and conformational, and dynamic properties of linear chain and branched melts are presented in Section 3. Section 4 concludes the paper with some general discussion of the significance of our results.

## 2. Model and Methodology

**Figure 1:**
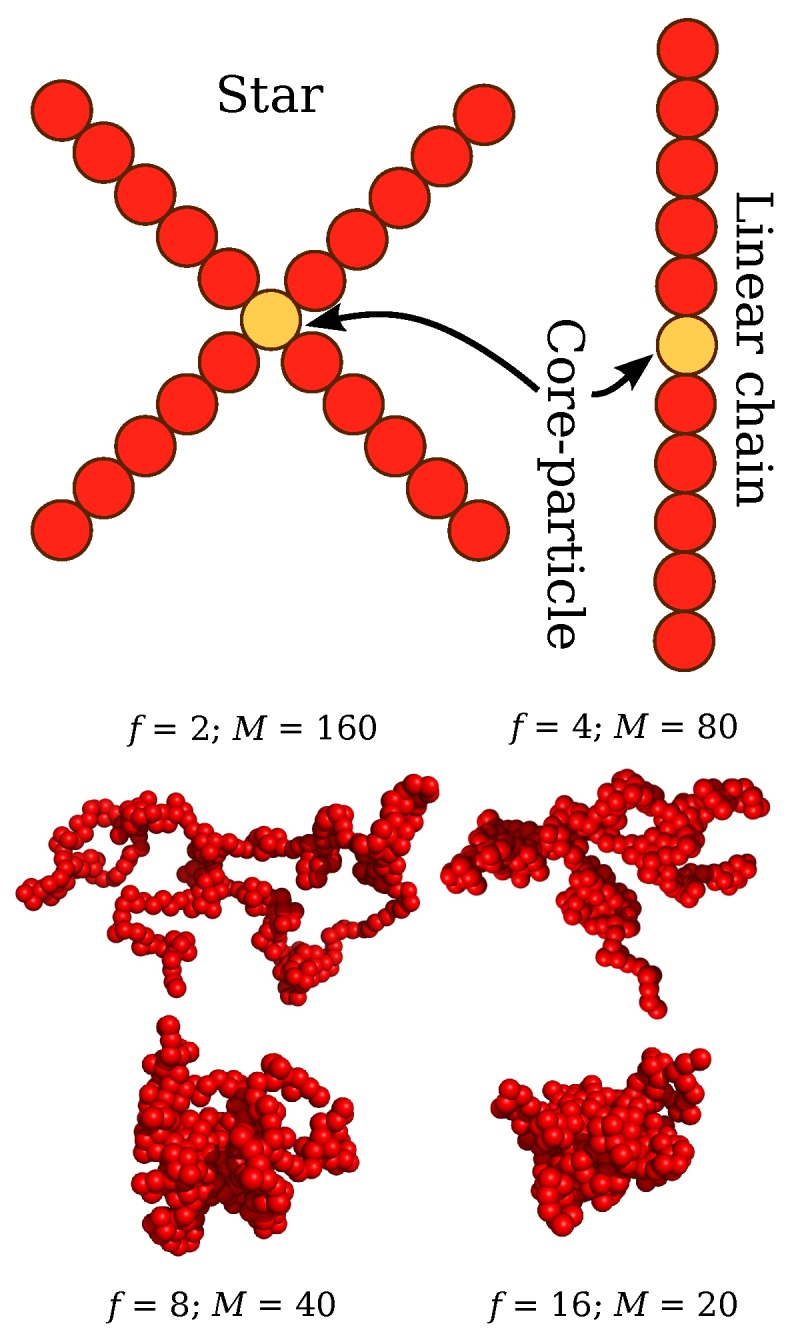
Schematic illustration of the topological architecture of regular stars, and linear chains. Screenshots of typical molecular conformations of polymers having different molecular architectures at the same molecular mass are also presented.

Our system consists of $N_{p}$ polymers with $N_{p}=400$. A star polymer is represented as a spherical core particle with *f* attached arms and each arm is composed of *M* segments with a total number of interaction centers $M_{w}=fM+1$. A linear chain is denoted as a star polymer with f=2 and its the core particle is taken to be the same type as those of the arms. The molecular parameters investigated correspond to arm lengths having a molecular mass $M_{w}=641$, 321, and 161 segments, which are above the estimated boundary between unentangled and entangled regimes for linear chains on a Lennard-Jones chain models, i.e., entanglement length $M_{e}\approx 85$ [40]. We examine four different functionalities, f=2, 4, 8, and 16. Additional molecular masses are considered that are in the unentangled regime ($M_{w}<M_{e}$) with $M_{w}=81$, 41, 21, and 11. The interactions between polymer segments are described by a cut-and-shifted Lennard-Jones (LJ) potential where ε and σ define the units of energy and length, and a cutoff distance $r_{c}=2.5$ σ. The core–core and core–monomer interactions are modeled as purely repulsive Weeks-Chandler-Andersen potential [41] with a modification taking into account the difference in the particle sizes [42]. The segments along a chain are connected with their neighbors via a stiff harmonic spring, $V_{H}\left(r\right)=k{\left(r-l_{0}\right)}^{2}$, where $l_{0}=0.99$ σ is the equilibrium length of the spring, and k=2500 $\varepsilon /\sigma^{2}$ is the spring constant. In terms of the units of real polymer chains, the beads should be identified with statistical segments of flexible polymer having a typical scale on the order of 1 nm to 2 nm [3] and the core particle of the stars should have a dimension on the order polymer monomer and we then take $R_{c}=0.5\;\sigma$ as representative estimate of the star core size. The energy and interaction range parameters are chosen to be the same for these interactions such that $\varepsilon_{\mathrm{cc}}=\varepsilon_{\mathrm{cb}}=\varepsilon$ and $\sigma_{\mathrm{cc}}=\sigma_{\mathrm{cb}}=\sigma$. Typical polymer conformations are presented in Figure 1.

Simulations were performed in a cubic box with length *L*; periodic boundary conditions were applied in all three directions. We utilized the large-scale atomic/molecular massively parallel simulator (LAMMPS) [43]. Simulations were performed in the NVT ensemble after equilibration in the NPT ensemble at the desired temperature. The time step was set to δt=0.005 τ, where $\tau =\sigma {\left(m_{b}/\varepsilon \right)}^{1/2}$ is the unit of time. Temperature and pressure are measured in units of $\varepsilon /k_{B}$ and $\sigma^{3}/\varepsilon$, respectively. Simulations were performed at different temperatures T=0.75 and 〈P〉≈0.1 in reduced units.

## 3. Results and Discussion

### 3.1. Packing Length

The reptation model and its generalizations do not provide a prediction of what molecular factors govern the “critical entanglement molecular mass”, $M_{c}$, at which the mass scaling of the viscosity, η, and polymer center of mass *D*, change from their low mass scaling of the unentangled regime to a new scaling relation for $M>M_{c}$. This phenomenology defines “entanglement”, whatever its physical origin. Experimental studies, however, have also indicated a strong structural correlation with between a closely related related quantity $M_{e}$, defined by the reciprocal of the plateau modulus [5] and the “packing length”, *p* [44,45,46,47]. $M_{c}$ differs from $M_{e}$ by roughly a factor of approximately 2 [5], and it is generally appreciated that $M_{e}$ and $M_{c}$ “track” each other even if these properties are not actually equivalent.

The packing length is defined [44,45,46,47] by the ratio of the volume occupied by the polymer divided by the square of the polymer radius of gyration, $R_{g}$, so that this quantity has units of length and its value is on the order of the statistical segment size,

(1) $$p=\frac{V_{\mathrm{occ}}}{R_{g}^{2}}=\frac{M_{w}}{R_{g}^{2}\rho }.$$

The volume occupied by the polymer is determined by $V_{\mathrm{occ}}=\frac{M_{w}}{\rho }$, where ρ is the segmental density. According to arguments noted above by Flory [6], and later supported by detailed theoretical modeling by Freed and Edwards [7] and others [48,49,50], the excluded volume interactions of linear polymer chains are screened at high segmental densities, leading the polymer chains to adopt configurations equivalent to random walk chains, also know as “Flory theorem”. Many experimental studies have established that $R_{g}∼M_{w}^{1/2}$, thus supporting the “Flory theorem” and this result widely viewed as triumph of modern polymer science. The implication of this result is that *p* is a *constant* for long flexible linear polymer chain melts and this basic configurational molecular property has been extensively tabulated for various polymers [46].

We emphasize that this asymptotic large mass scaling of $R_{g}∼M_{w}^{1/2}$ has only been established for *linear polymer chains*. Recent simulation and experimental work has indicated that $R_{g}$ for unknotted rings scales with polymer mass with a power of approximately 1/3 with increasing polymer mass [51,52,53]. This scaling would imply that *p* should diverge to infinity as $M_{w}\to \infty$ for polymers having this non-linear molecular topology. This increase suggests that pure melts of this type of polymer should never become entangled, as in the case of linear chains. We are then led to consider how *p* varies with branching topology. We also note that increasing chain stiffness has the effect of increasing $R_{g}$ of polymers, which tends to reduce *p*, suggesting that stiffer polymers should more readily “entangle” at a given mass based on this entanglement criterion. Experimental evidence supports this trend with chain rigidity, raising questions regarding the role of polymer knotting in the chain “entanglement” phenomenon [54].

**Figure 2:**
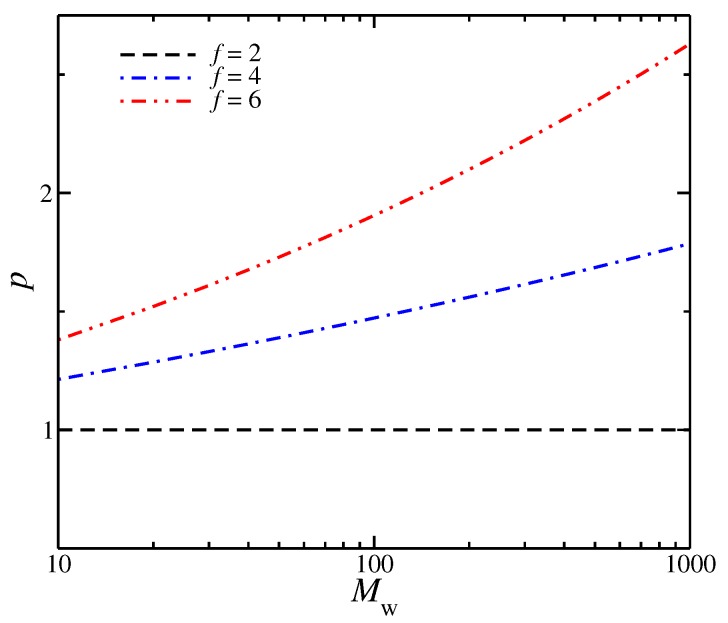
Packing length, *p* as a function of molecular mass, $M_{w}$; segmental density is assumed ρ=1. A progressive increase of *p* with *M* and diminished entanglement is also expected in bottlebrush polymers based on the $R_{g}$ mass scaling observed in [59].

Recent simulation studies have suggested that other molecular architectures than linear chains may have mass scaling exponents governing polymer size (e.g., $R_{g}$), less than ν=1/2. For randomly branched polymers in their melt state, ν has been proposed to be exactly 1/3 [55,56,57,58], indicating that these polymers also form rather ‘compact’ structures in the melt state [59]. Ring polymers in the melt have been predicted to exhibit this same type of asymptotic scaling [52], strongly suggesting that these polymers belong to the randomly branched polymer universality class when they are in the melt state. Screening evidently operates differently between linear chains and randomly branched polymers. In recent work, the authors found that both regular branched polymers, i.e., stars and unknotted rings [60], and bottlebrush polymers [59], exhibit a scaling of $R_{g}$ with *M* over a wide range of *M* that is more similar to randomly branched polymers and rings than linear polymer melts, i.e., ν is significantly less than the random walk value of 1/2. We reiterate that an exponent ν<1/2 for polymers in the melt state means that *p* should progressively increase with $M_{w}$ for all these non-linear polymer melts. Indeed, an increase in *p* is apparent in all our simulated branched polymer melts, in contrast, *p* remains constant for linear chain melts; see Figure 2. Correspondingly, we expect a diminished tendency of branched polymers to entangle by the packing length criterion.

We mention that the mathematical equivalent of *p* for polymers arises in many other areas of physics and mathematics. Specifically, the ratio of the volume swept out by an ideal Brownian particle divided by its mean square radius of gyration of its trajectory defines the “capacity”, *C*, of the particle [61]. Recent work has shown that *C* is essentially equivalent to the particle hydrodynamic radius $R_{h}$ and this quantity has many other applications (it is proportional to the Smoluchowki diffusion-limited rate constant, etc., self-electrostatic capacity, scattering lengths in acoustics and quantum theory, etc.) [61]. In a lattice model context, this quantity corresponds to the number of sites visited by a random walk divided by $R_{g}^{2}$ and this ratio $C^{*}$ is exactly related to the average number of intersections of long random walk, and many of the critical constants of statistical mechanics (percolation thresholds, critical temperatures of spin models, critical binding energies for particle localization, etc.) can be approximately expressed in terms of $C^{*}$ [62]. It is no wonder that *p* has served as a useful quantity in understanding the packing of entangled polymer chains [63] and the thermodynamics of polymer blend miscibility [64], since the capacity governs the contact probability of random coil polymers [62].

### 3.2. Influence of Polymer Mass and Scaling of $R_{g}$

**Figure 3:**
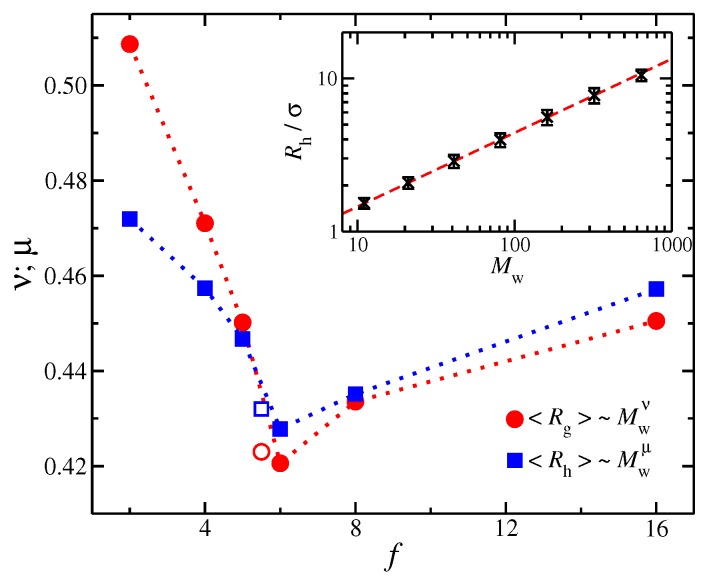
The mass scaling exponents ν (circles) and μ (squares) for radius of gyration $R_{g}$ and $R_{h}$, respectively, as the function of functionality, *f*; filled symbols correspond to the linear chain (with f=2) and star polymers and open symbols to ring polymers. Inset: $R_{h}$ as a function of molecular mass $M_{w}$ for linear chains.

As discussed above, the size of a polymer can be described by $R_{g}$, which typically scales with $M_{w}$ as a power-law, i.e., $R_{g}∼M_{w}^{\nu }$. The mass scaling of polymers have been extensively studied in the past both in solution and in the melt [1,2,3]. Based on our previous study of regular stars in the melt state [38], we again find that the effective ν exponent indicates a relatively compact configuration relative to random coil polymers, i.e., ν<1/2, see Figure 3. As the star functionality increases, ν progressively decreases and then reaches a minimum value at f=6 where ν takes a value approximately equal to the effective value having unknotted ring polymers for comparable $M_{w}$. For f>6, there is small increase in ν due to the stretching of the chains near to the core of the particle. Our previous findings are based on polymers having $M_{w}<10\;M_{e}$, so that it remains an open question as to what values these apparent exponents take in the limit, $M_{w}\to \infty$.

Another important length characteristic of polymers is the hydrodynamic radius $R_{h}$, which is a function of polymer conformation in hydrodynamic theory. We find that this quantity qualitatively follows the same mass scaling trends as with $R_{g}$ for linear polymer chains. A typical example of mass scaling with $R_{h}$ for linear polymers is presented in Figure 3 where the mass scaling exponent is indicated to equal, 0.48. We discuss the importance of $R_{h}$ for the determination of *D* below for both linear and branched polymers.

### 3.3. Quantification of the Influence of Molecular Topology on Molecular Packing

**Figure 4:**
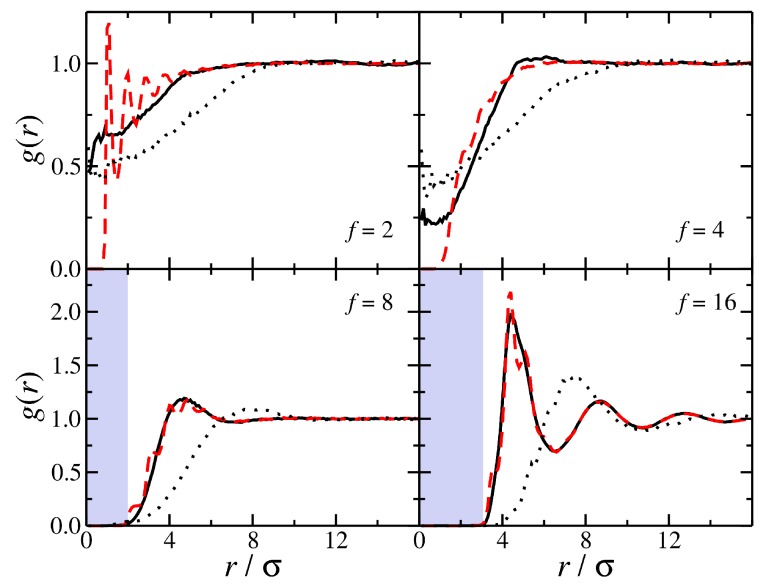
Comparison of the radial distribution function g(r) of the polymer center-of-mass (black continuous line) and the core particle (red dashed line) for polymers having molecular mass, $M_{w}=81$. Results for different functionalities and the g(r) for the polymer center of mass of polymers having $M_{w}=321$ (black dotted line) are also presented. The highlighted regions illustrate the emergent particle-like character of the polymer.

Branching clearly results in the formation of more compact polymer conformations than linear chains, which correspondingly influences the packing of the polymers in the melt. Typically the packing of the polymers is described by the structural correlation functions, such as radial distribution function, g(r). For linear chains, the g(r) of the polymer center of mass exhibits characteristics of ultra soft particles where the center of mass of two polymers can overlap [65], i.e., g(r)>0 for r/σ<1. We note that the g(r) for the core particle and the g(r) for the polymer center of mass are distinct at length scales smaller than the polymer size. Increasing the molecular mass of linear chains increases the depletion region $r/\sigma <R_{g}$ in the g(r) for polymer center of mass. As *f* increases, particle-like correlations emerge between star polymers [66,67,68]. The probability for the polymer center of mass at short distances decreases and for highly branched stars we see that there no overlap region in the polymer center of mass in g(r) when *f* becomes sufficiently large; see Figure 4. Moreover, g(r) of the polymer center of mass and that of the core particle become approximately the same, see Figure 4. This means that the center of mass and the core particle essentially coincide in space in an average sense for highly branched star polymers [68]; an effect also seen in polymer grafted nanoparticles [69,70]. We can view this effect as a kind of the core particle “localization” that accompanies the emergence of the particle-like character of the polymers.

**Figure 5:**
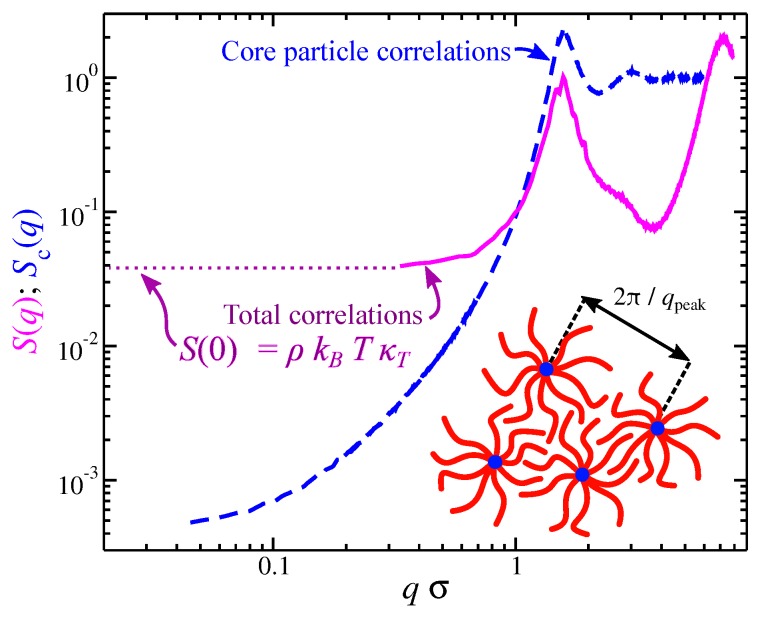
Total static structure factor S(q) (continuous line) and partial structure factor of star polymer melts. The relation between the $S\left(0\right)=\rho \;k_{B}T\;\kappa_{T}$ for S(q) and a schematic of the packing for star polymers are also shown, Reproduced with permission from [73]. Copyright American Physical Society, 2018.

Polymers near this transition between random coil to particle-like conformational structure exhibit strong fluctuations in shape that can greatly impact molecular segmental packing. Segmental packing at a local scale is subject to thermal fluctuations which influence the density fluctuations at large scales. This coupling can be understood from the structure factor S(q) (the Fourier transform of g(r)). As seen in Figure 5 the structure factor between the segmental and core particle are quite distinct. Of particular importance is S(q) in the limit where the wave vector *q* goes to zero. Specifically, we have $S\left(0\right)=\rho kT\kappa_{T}$ at equilibrium, where ρ is the segmental density and $\kappa_{T}$ is the isothermal compressibility defined as $\kappa_{T}=-\frac{1}{V}{\left(\frac{\partial V}{\partial P}\right)}_{T}$. The segmental packing for polymer melts result in values of S(0) that are small in comparison to hard particles, but the core particles and, by extension of the above discussion, the polymer center of mass, exhibit anomalous small density fluctuations, a feature known as “hyperuniformity” [71,72]. This large suppression of density fluctuations in these floppy molecules is associated with the localization of the core particle within the polymeric structure and renders branched polymers as candidates for hyperuniform materials [70,73]. These results illustrate the importance of understanding molecular packing in material properties.

These structural correlations suggest that polymers as a whole can be viewed as “soft” particles having a variable degree of overlap with neighboring polymers, depending on molecular topology, stiffness, etc. [65,74,75,76,77,78] This coarse-grained perspective suggests that it is possible to develop models for polymer transport in the melt that do not rely on specific molecular topology, giving hope for the development of a unifying perspective of polymer melt dynamics that is agnostic regarding the role polymer topology in the process of molecular diffusion. Treating polymers as “soft” particles is an old idea, for example Flory and Krigbaum [79] modeled polymers in solution by a mean field Gaussian segmental density cloud. Gobush et al. [80] generalized this picture to more faithfully reflect the average anisotropic shape of flexible polymers. The Gaussian segmental cloud description of polymers has recently reemerged in coarse-graining studies of the thermodynamic and dynamic properties of polymer melts [65,74,75,76,77,78].

### 3.4. Application of Stokes-Einstein and Fractional Stokes Einstein Relations to Polymer Melts

There is a long history of treating molecular diffusion in liquids through the Stoke-Einstein fluctuation-dissipation relation between *D*, thermal energy $k_{B}T$, the fluid viscosity η, and the hydrodynamic radius, $R_{h}$. While $R_{h}$ is normally measured in solution, it is certainly possible to measure the tracer diffusion coefficient *D* of polymers in the melt [81,82,83,84] and the determination of *D* in the melt is particularly natural for molecular dynamics simulations.

Now, if we assume and take $\eta ∼R_{g}^{2}∼M_{w}$, in accord with Rouse model and experimental reports, then in the view of approximately scaling $R_{h}∼R_{g}$ for linear flexible polymers, we may expect *D* to scale as $D∼k_{B}T/R_{h}^{3}$ for linear chains; we note that above calculations are approximate, since $R_{h}/R_{g}$ reaches a plateau for $M_{w}\gg M_{e}$ [13,38]. Despite the shortcomings of the Rouse model, recent simulations by Xu et al. [85] and experiment seem to support the scaling $\eta ∼R_{g}^{2}$ for star polymers so that an inverse scaling of *D* with $R_{g}$ with a power near −3 is also plausible for branched polymers. We previously found this scaling to be a good approximation for linear and branched polymer unentangled polymer melts. However, Martin and coworkers [86,87] previously suggested that this scaling should have a somewhat modified form, $D∼k_{B}T/R_{h}^{2.7}$ for randomly branched polymers. In the limit of an extremely high degree of branching, where the polymers become ball-like, we must recover Stokes law, $D∼k_{B}T/R_{h}$, so a progressive reduction in the magnitude of the scaling exponent can be expected with increased branching density. In particular, star polymers have been shown to exhibit a transition to particle like behavior in the limit of many arms, consistent with the observation of the Stokes-Einstein scaling, $D∼k_{B}T/R_{h}$, for a large number of arms *f* [20,88,89]. We observed a trend in this direction in our previous work, i.e., $D∼R_{h}^{-\lambda }$ for a wide range of unentangled polymers from linear chains, stars, and unknotted ring polymers. Interestingly, we find the exponent λ has values of λ≈2.7 for lightly branched polymers [38], according with the estimation of Martin and coworkers for concentrated randomly branched polymers formed by cross-linking low molecular mass polymers [86,87].

This scaling of *D* with polymer size has also been rationalized by Wyart and DeGennes [90], and others following them [91,92,93,94], as arising from the particles “sensing” a local viscosity distinct from the macroscopic viscosity. These observations again suggest that we the consider diffusion of polymers in the melt as being similar in a coarse-grained sense to a tracer particle diffusion of particles having dimensions a comparable to the surrounding polymers. Consistent with this picture, we previously showed that the the λ=3 for linear polymer chains could be recovered from simulations of spheres having a size equal to the chain $R_{g}$ and identifying the spheres with a typical tracer “particle”, supporting this physical picture of the origin of λ [38]. However, our previous study [70] was restricted to unentangled polymers, and it is natural to extend our calculations to *entangled* polymers.

The basic premise of our treatment of the entangled regime regime is based on the general tendency of soft sphere fluids to form glass-forming liquids at high concentrations. Once the polymers are considered to be soft spheres (or ellipsoids), it is a natural proposition to consider entanglement to correspond to a type of entropically driven glass-formation [54,63,70,95]. There is direct evidence for dynamic heterogeneity in entangled polymer melts evidenced in recent polymer tracking measurements [96]. The role of molecular shape in the anisotropy in the case of melts of entanglement of linear polymers can also be expected to be important and it was previously suggested that these materials should form “nematic glasses” with an Onsager condition describing the intermolecular coupling, leading to a packing length criterion for the critical entanglement molecular mass, $M_{c}$ [63].

**Figure 6:**
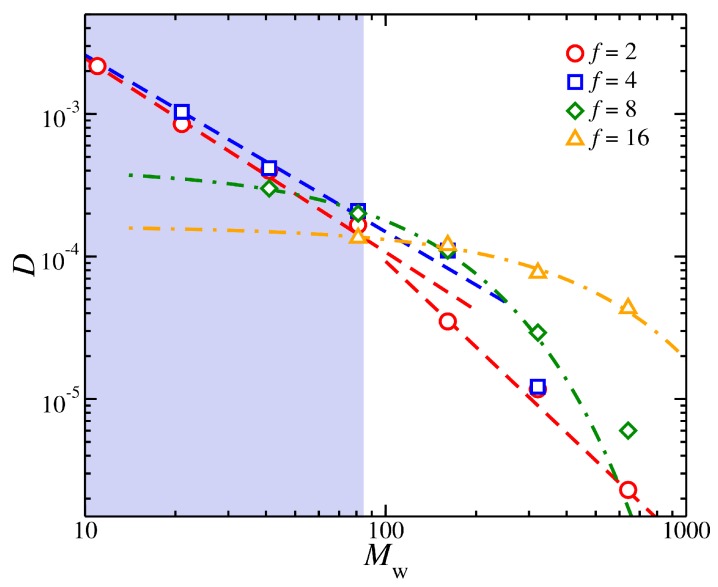
Self-diffusion coefficient *D* of the polymer center of mass as a function of the molecular mass, $M_{w}$, at temperature T=0.75. The highlighted region outlines the unentangled regime for linear chains. The dashed lines are guides for the eye and the dot-dashed lines are fits to an exponential relation, $D=\alpha \exp(-\beta M_{w})$, where α and β are fitting parameters.

We present our results for *D* as a function of $M_{w}$ in Figure 6. For linear chains, we clearly obtain a crossover from an unentangled to an entangled regime at $M_{w}\approx 85=M_{e}$, as found in previous work [40]. In the entangled regime, *D* scales approximately as $D∼M_{w}^{-2}$, which is consistent with experimental observations [97]. For highly branched polymers, i.e., f=8 and 16, we find that *D* can be better described by an exponential function rather than a power-law function. On the other hand, it has been observed previously that the viscosity of star polymer melts scales exponentially with arm mass, i.e., $\eta ∼\exp\left(M/M_{e}\right)$, over a wide range of functionalities, 2<f<33 [89]. This scaling was rationalized by de Gennes as arising from arm retraction mechanism of the stars. In particular, it was argued that in order for the core particle of the star to relax it must to wait for the arms to relax through retraction several times. Several intuition-based modeling studies have been made to adjust the star arm-retraction model to better fit experimental observations. We next develop a conceptually different model of the melt dynamics of stars.

Specifically, we start from a consideration of *D* as a function of $R_{h}$. In the unentangled regime $D∼R_{h}^{-\lambda }$, where λ≈2.7 was found to be a satisfactory description for linear chains and low *f* stars in a previous study [38]. We assume that in the entangled regime there is dynamic cluster formation, as in of glass-forming liquids, persisting on sufficiently long time scales and to dominate the stress relaxation in the fluid [95]. This physical picture of “entanglement” naturally leads us to expect a “decoupling” or “fractional Stokes-Einstein relation” between *D* and η as often found in glass-forming liquids, i.e., $D∼\eta^{-\delta }$, where δ<1 [95]. Data summarized by Wang et al. [98] indicates that δ is about 0.71 for a number of different entangled polymer melts so there is clear evidence consistent with entanglement giving rise to “decoupling” relation between *D* and η, a basic feature of glass-forming liquids. This view of entanglement is further supported by other evidence such a stretched exponential relaxation, aging and many other established features of glass-forming liquids [54].

If a transition to heterogeneous polymer dynamics on the scale of $R_{g}$ underlies the entanglement phenomenon in polymer melts, then we would expect a *transition* between $\eta ∼R_{h}^{\lambda }$ to $\eta ∼R_{h}^{\lambda /\delta }$ as the polymer enters the dynamically heterogeneous melt regime. We indeed obtain $D∼R_{h}^{-\lambda /\delta }$ in the entangled polymer melt regime, as anticipated. In particular, δ in our simulations is found to be about δ≈2/3; see Figure 6. This estimate of the decoupling exponent δ is typical of glass-forming liquids where this type of fractional power-law relating η and *D* is referred to as the “fractional Stokes Einstein relation” [99]. Since $R_{h}∼M_{w}^{\mu }$ with μ≈0.485 for linear chains, then we get $D∼R_{h}^{-\lambda }∼M_{w}^{-\lambda \mu }∼M_{w}^{-2.1}$, according with experimental observations [97] of the mass scaling exponent of *D* in the entangled regime $D∼M_{w}^{-2.3}$ [100]. Similar results are obtained from f=4 stars, but for highly branched stars we find that the *M* variation of *D* switches to an exponential form, $D∼\exp\left(-R_{h}\right)$, that is more similar to an exponential $M_{w}$ dependence, as noted above. Evidently, some other factor must be important for understanding the dynamics of branched polymers.

We take the view that the observed exponential dependence of *D* on the polymer mass in stars is a reminder that the polymer topology also alters the *thermodynamics* of polymer melts. Polymers are *molecules* rather than macroscopic particles where hydrodynamics obviously applies. In particular, the activation energy governing *D* and η of polymer melts, at least at high *T*, when the glassy dynamics is not prevalent, is dominated by the cohesive energy density of the fluid [13,101]. A change in the polymer topology can be expected to alter the cohesive interaction strength, and thus the activation energy for transport properties, an aspect of fluid dynamics that is not captured by a purely hydrodynamic description. We then interpret the exponential variation of *D* to naturally arise from a change of activation energy due to a change in molecular topology.

We start our consideration by recognizing that recent studies of transport of particles in concentrated polymer fluids have indicated an apparent *D* of the tracer particles that exhibits an apparently universal scaling, $D∼\exp\left(-E_{a}/k_{B}T\right)$, where the activation energy $E_{a}$ scales as a power of the particle radius, $E_{a}∼R_{h}^{\theta }$, where the power θ is often found to be empirically near one [102]. Based on the arguments presented in the discussion above, we consider a *hybrid* expression for *D* that addresses both hydrodynamic and thermodynamic effects of the altering chain topology on *D*,

(2) $$D=\alpha \;R_{h}^{-\lambda /\delta }\;\exp\left(-R_{h}/\gamma \right).$$

**Figure 7:**
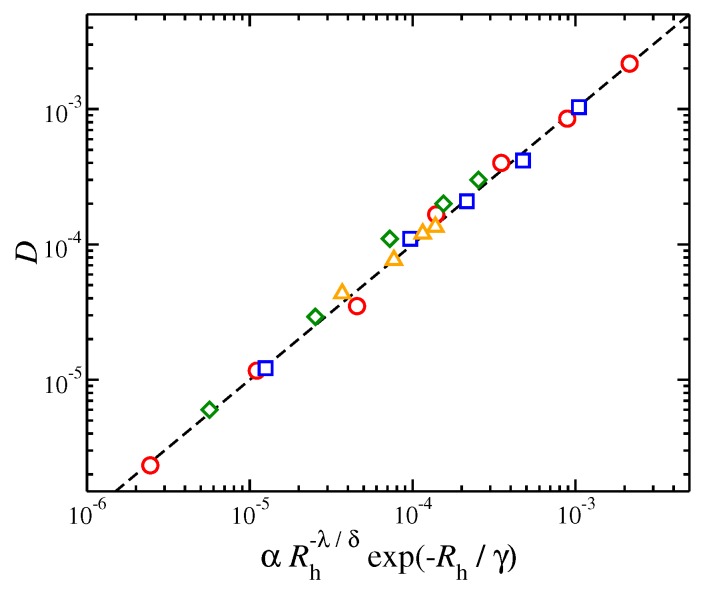
Self-diffusion coefficient of the polymer center of mass, *D*, as a function of a function form based on hydrodynamic radius, $R_{h}$. Results for entangled and non-entangled polymers as well as polymers of different functionality, *f* are also presented. The values of the parameters α, λ, γ, and δ. The symbols are the same as in Figure 6. The dashed line is a guide for the eye.

The prefactor α is a fitting parameter that appears to obey the scaling relation of $\alpha \approx f^{-5/2}$ for star polymers when $M<M_{c}$; for linear polymers an additional factor is necessary, i.e., $\alpha \approx \frac{1}{24}f^{-5/2}$ for unentangled and $\alpha \approx \frac{5}{12}f^{-5/2}$. The parameter γ describes a crossover from “soft” linear chains to particle-like highly branched stars in the limit, f→∞. For highly branched stars, which exhibit particle-like characteristics, γ is found to be of the order of unity. The decoupling exponent δ represents the crossover from unentagled to the entangled regimes, as described above. Specifically, for unentangled systems we have δ=1 and while because of “decoupling” we have δ<1 for entangled melts. The values for these parameters and exponents for each case are presented in Table 1 and the quality of agreement between the calculated values of *D* and the proposed function form based on $R_{h}$ in Equation (2) is presented in Figure 7.

## 4. Conclusions

In summary, we investigated the packing and conformational properties of entangled polymers and their dynamics with the use of a coarse-grained polymer model. In particular, we calculated the self-diffusion coefficient of the polymer center of mass, *D*, and the hydrodynamic radius $R_{h}$ for linear chain in unentangled and entangled regimes, as well as, regular stars at equivalent molecular masses in the melt state. We utilized a path-integration algorithm, ZENO, to calculate the hydrodynamic radius $R_{h}$ of the polymers in the melt state. We find that we can rationalize the dependence of *D* based on the polymer $R_{h}$ by viewing polymers in the melt as being similar to “particles” whose degree of “softness” is influenced by their molecular topology. Specifically, we develop an empirical relation for the self-diffusion coefficient of the polymer center of mass for polymer in the melt state, which describes *D* in both the entangled and unentangled regimes. This relation also accords with *D* data for high branched star polymers. Our approach provide a provides a tentative unifying framework that is agnostic to the polymer topology (e.g., linear chain, star, bottlebrush, and ring), thus offering a practical approach for describing diffusion and viscosity of polymer melts having different molecular architectures.

## Figures and Tables

**Table 1:** List of parameters of Equation (2) for polymers of varying functionality *f* and arm length *M*.

*f*	*M*	α	γ	δ	λ
2	$<M_{e}/2$	$\frac{1}{24}f^{-5/2}$	∞	1	2.88
2	$>M_{e}/2$	$\frac{5}{12}f^{-5/2}$	∞	2/3	2.88
4	$<M_{e}$	$\frac{1}{6}f^{-5/2}$	∞	1	2.42
4	$≳M_{e}$	0.5	2.2	2/3	2.42
8	−	$\frac{1}{6}f^{-5/2}$	1.60	1	−0.46
16	−	$\frac{1}{6}f^{-5/2}$	1.95	1	−1.25
